# PIK3CA mutations and PI3K isoform expression define prognostic signatures in ovarian clear cell carcinoma

**DOI:** 10.1007/s00404-026-08525-w

**Published:** 2026-07-14

**Authors:** Xiaonan Zhou, Yifei Liu, Jing Zhang, Min Ren, Gang Ji, Xu Cai, Rui Bi

**Affiliations:** Department of Pathology, Fudan University Shanghai Cancer Center, Fudan University, 270 Dong’an Road, Shanghai, 200032 China

**Keywords:** Ovarian clear cell carcinoma, *PIK3CA* mutations, PI3K subunits, Prognosis

## Abstract

**Purpose:**

Ovarian clear cell carcinoma (OCCC) is frequently characterized by dysregulation of the PI3K/AKT/mTOR (PAM) pathway; however, the expression patterns and clinical significance of class I PI3K catalytic isoforms remain incompletely understood. This study aimed to comprehensively evaluate PI3K catalytic subunit expression, co-expression patterns, and their prognostic relevance in OCCC.

**Methods:**

A retrospective cohort of 100 patients with OCCC was analyzed. Immunohistochemistry was performed to assess the expression of p110α, p110β, p110δ, p110γ, and pAKT. PIK3CA exon 9 and exon 20 mutations were evaluated in available formalin-fixed paraffin-embedded tissues. Associations between PI3K isoforms, pAKT activation, clinicopathological characteristics, and survival outcomes were investigated.

**Results:**

The expression positivity rates of the four class I PI3K catalytic subunits in OCCC were 86% for p110γ, 77% for p110α, 58% for p110δ, and 56% for p110β. p110α and p110β were positively associated with pAKT activation, whereas p110δ and p110γ showed no significant association. p110δ expression was significantly correlated with PI3KCA exon9 mutation. Frequent co-expression of multiple PI3K catalytic subunits was observed, with simultaneous expression of all four isoforms representing the predominant pattern and significantly correlating with pAKT positivity. In advanced-stage disease, p110β expression was significantly associated with poorer progression-free survival (PFS), while pAKT positivity correlated with worse overall survival (OS) and PFS.

**Conclusions:**

OCCC displays substantial heterogeneity in PI3K catalytic isoform expression and co-expression. p110α and p110β were associated with pAKT activation. In advanced-stage OCCC, high p110β expression was associated with poorer PFS, whereas pAKT activation was associated with both poorer PFS and OS, supporting further investigation of PI3K-targeted therapeutic strategies.

**Supplementary Information:**

The online version contains supplementary material available at https://doi.org/10.1007/s00404-026-08525-w.

## Introduction

Ovarian clear cell carcinoma (OCCC) is characterized by a variety of molecular abnormalities. Among them, the mutation rate of the *ARID1A* gene is approximately 50%, while that of the *PIK3CA* gene ranges from 30 to 40% [1, 2]. Notably, *PIK3CA* mutations often co-occur with *ARID1A* mutations and are considered early molecular events in the disease process. Studies have demonstrated that coexistence of *ARID1A* and *PIK3CA* mutations can drive tumorigenesis [1], with the loss of *ARID1A* may lead to chromatin remodeling defects that could enhance the activation of the PI3K-AKT-mTOR (PAM) pathway by *PIK3CA* mutations [2]. In addition, other low-frequency mutations, including *PTEN*, *TP53*, *TERT*, *KRAS*, and *BRCA2* have also been observed [2, 3]. These genetic alterations ultimately trigger the abnormal activation of the PAM pathway as a key mechanism in OCCC.

The PAM pathway is critical for regulating cell proliferation, survival, and metabolism, and its dysregulation is common in many cancers. This pathway is frequently activated by mechanisms such as *PIK3CA* mutations, *PTEN* loss, and *PIK3CA* amplifications [4]. The catalytic subunits of PI3K, especially the Class I isoforms (p110α, p110β, p110δ, and p110γ), play a crucial role in this activation. However, the role of different PI3K catalytic subunits in OCCC remain unclear.

This study aims to explore the expression and prognostic value of PI3K catalytic subunits in OCCC, and their associations with clinical outcomes, thereby providing a theoretical basis for targeted therapeutic strategies.

## Method

### Patients and tissue samples

Patients were included in this study based on the following criteria: (1) patients diagnosed with primary OCCC who underwent surgical resection at Fudan University Shanghai Cancer Center (FUSCC) between 2000 and 2015; and (2) availability of tumor specimens suitable for tissue microarray (TMA) construction. Clinical data extracted from medical records included age, tumor size, laterality, presence of ascites, preoperative serum CA125 levels, and International Federation of Gynecology and Obstetrics (FIGO) stage, which was reclassified according to the 2014 FIGO guidelines [5]. We also evaluated the presence of endometriosis (ovarian and/or extra-ovarian) in the specimens. Follow-up data were collected through medical records or telephone interviews until May 2023.

### Tissue microarray (TMA) construction and immunohistochemistry

TMAs were constructed using 1-mm triplicate core samples from 108 OCCC specimens, while the remaining 8 cases were excluded due to tissue loss from the tissue microarray. Immunohistochemical staining was performed on the TMAs following previously described protocols [6], using antibodies against p110α, p110β, p110δ, p110γ, and pAKT (Supplementary Table 2). All H&E and IHC slides were reviewed by Dr. Bi. PI3K catalytic subunits and pAKT expression were semi-quantitatively scored (0–3 +) according to staining intensity. Cytoplasmic positivity was defined as immunoreactivity in ≥ 10% of tumor cells within the tissue cores. Considering the intratumoral heterogeneity within TMAs, the average score of 3 TMA cores was used as the final score. Immunohistochemical scores of 2 + and 3 + were considered positive [7].

### DNA extraction and Sanger sequencing

Genomic DNA was extracted from all formalin-fixed paraffin-embedded (FFPE) tissue samples using the QIAGEN DNA FFPE Tissue Kit (Qiagen, Shanghai, China). DNA quality and yield were assessed using a NanoDrop Spectrophotometer (Thermo Fisher Scientific, Shanghai, China). Sanger DNA sequencing was performed by Sangon Biotech Co., Ltd. (Shanghai, China), and the sequencing data was analyzed using SnapGene version 6.0.2 software. All detected mutations were confirmed by resequencing. The primers used for *PIK3CA* exons 9 and 20 are listed in Supplementary Table 3.

### Statistical analysis

Statistical analyses were performed to identify correlations between p110α, p110β, p110δ, p110γ, pAKT and other clinicopathologic factors using the χ2 test or Fisher’s exact test, as appropriate. To account for multiple testing in the association analyses, P-values were adjusted using the Benjamini–Hochberg false discovery rate (FDR) procedure, and p-value < 0.05 was considered statistically significant. OS and PFS were analyzed using the Kaplan–Meier method, and differences between survival curves were evaluated using the log-rank test. The Cox proportional hazards regression model was used to investigate the prognostic significance of clinicopathologic factors. To identify independent prognostic factors, all significant variables in the univariate Cox regression analysis (P < 0.05) were subjected to multivariate Cox regression analysis. Hazard ratios (HRs) and 95% confidence intervals (CIs) were calculated. For survival analyses, a two-sided p-value  < 0.05 was considered statistically significant. All statistical analyses were performed using SPSS v25 and R v4.2.2.

## Results

### PI3K catalytic subunit expression and *PIK3CA* mutational profiles in OCCC

The median age was 54 years (range, 28–83 years), and the median maximum tumor diameter was 11 cm (range, 2–27 cm). A total of 69% (69/100) of patients were classified as FIGO stage I or II, and 79% (79/100) had unilateral disease. Endometriosis was observed in 36% (36/100) of ovarian and/or extra-ovarian specimens, adenofibroma was present in 5% (5/97) of patients, and ascites was observed in 47% (47/100) of cases. Elevated preoperative serum CA125 levels were observed in 78% (64/82) of patients. The clinicopathological characteristics of the study cohort are provided in Supplementary Table 1.

**Fig. 1:**
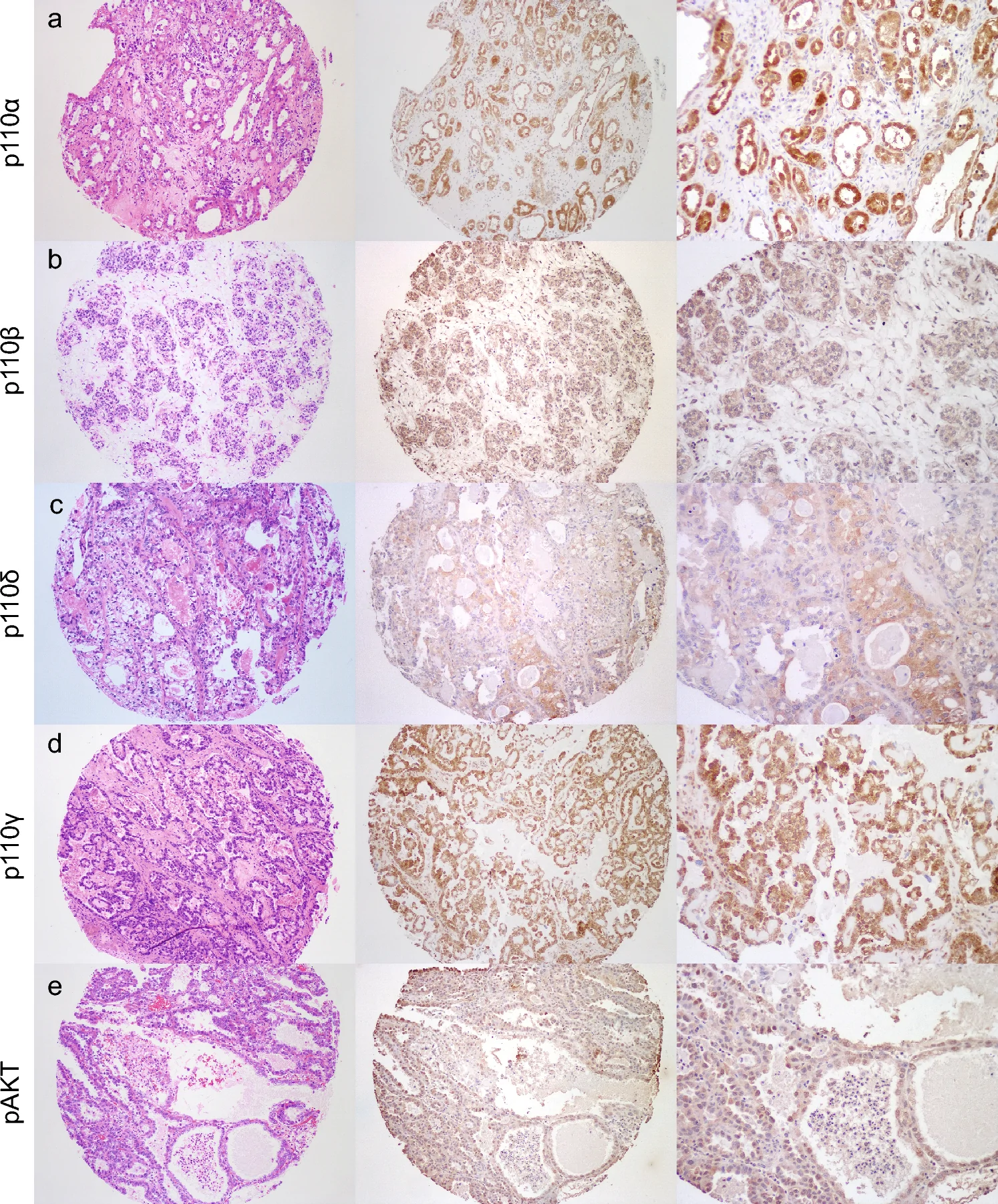
**a** Immunohistochemical staining of p110α; **b** Immunohistochemical stain for p110β; **c** Immunohistochemical stain for p110δ; **d** Immunohistochemical stain for p110γ; **e** Immunohistochemical stain for pAKT. The images were captured at 40 × magnification

Immunohistochemical analysis of class I PI3K catalytic subunits and pAKT was successfully performed in all cases. Among the four PI3K catalytic isoforms, p110γ was the most frequently expressed subunit, detected in 86% of tumors, followed by p110α (77%), p110δ (58%), and p110β (56%). In addition, pAKT positivity was observed in 58% of tumors, indicating frequent activation of downstream PI3K signaling in OCCC. Representative immunohistochemical staining images are shown in Fig. 1a–d.

Due to prolonged storage of FFPE tissue blocks, sequencing of *PIK3CA* exon 9 was successfully completed in 75 cases, and exon 20 in 77 cases. To evaluate the potential for selection bias resulting from incomplete PIK3CA sequencing, clinicopathological characteristics were compared between the sequenced (n = 77) and non-sequenced (n = 23) cohorts. No significant differences were observed between the two groups (Supplementary Table 4), suggesting that unsuccessful sequencing due to FFPE degradation was unlikely to introduce substantial selection bias.

**Fig. 2:**
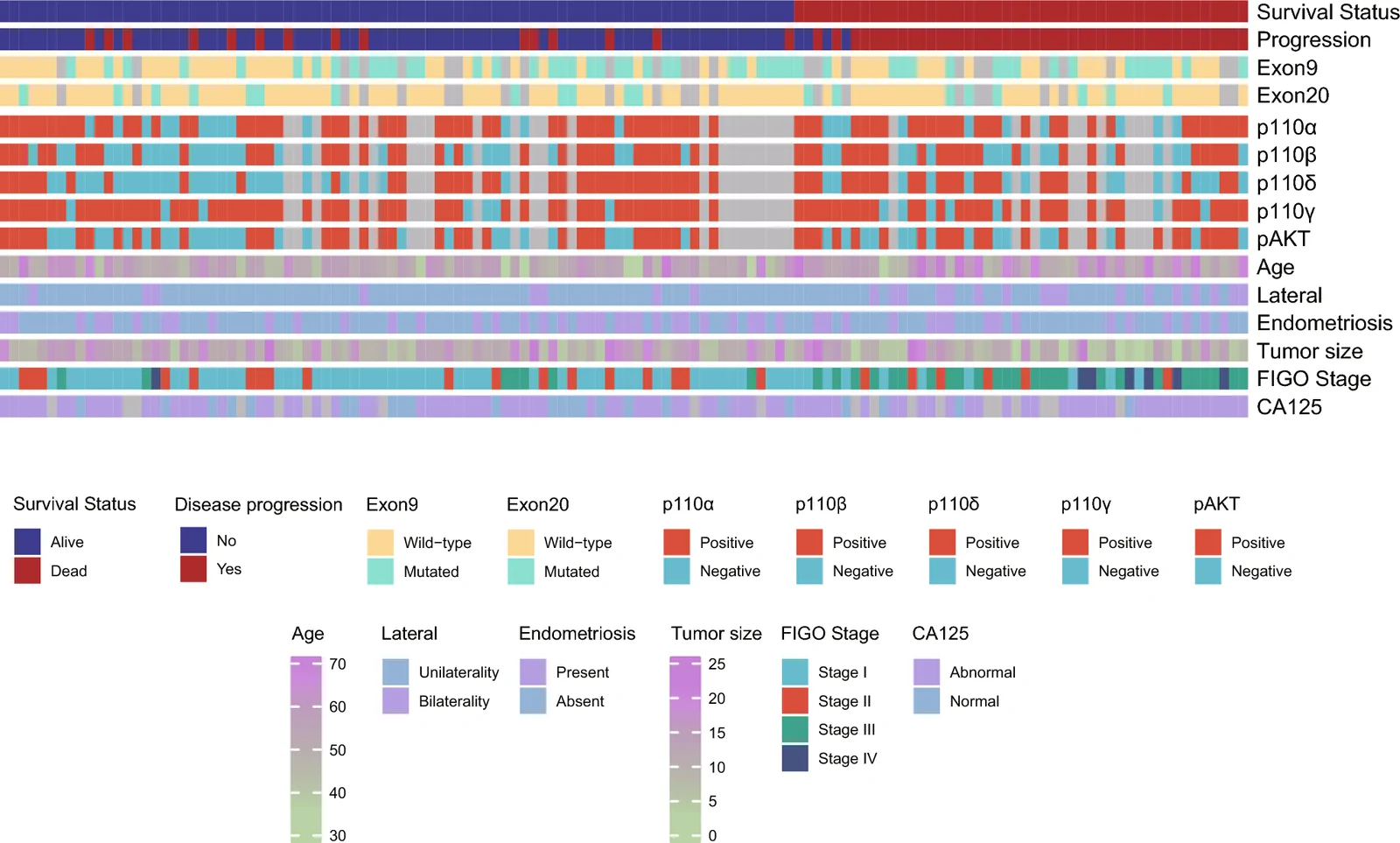
Heatmap of Clinicopathological Factors, PI3K catalytic subunits and pAKT expression

DNA sequence analysis of exons 9 and 20 of the *PIK3CA* gene revealed mutations in 52% (40/77) of OCCC patients. Specifically, mutations in exon 9 were present in 39% (29/75) of tumors, while exon 20 mutations were detected in 21% (16/77). Co-mutations in both exons were observed in 6.5% (5/77) of patients. A heatmap integrating patient survival statuses with clinicopathological features, PI3K catalytic subunit expression, and *PIK3CA* mutational profiles is shown in Fig. 2.

### Association between class I PI3K catalytic subunits expression and clinicopathological characteristics

To investigate the biological relevance of PI3K signaling activation in OCCC, we analyzed the relationship between PI3K catalytic subunit expression and pAKT activation. pAKT positivity was significantly associated with increased expression of p110α and p110β, whereas no significant associations were observed with p110δ or p110γ expression. In addition, p110δ expression was significantly correlated with PIK3CA exon 9 mutations (p = 0.035, Table 1), suggesting a potential functional link between genomic alterations and downstream pathway activation.

**Table 1:** The association between the catalytic subunits of the class I PI3Ks and clinicopathological factors

	p110α		P	p110β		P	p110δ		P	p110γ		P	pAKT		P
	Negative(%)	Positive(%)		Negative(%)	Positive(%)		Negative(%)	Positive(%)		Negative(%)	Positive(%)		Negative(%)	Positive(%)	
Age			1.000			1.000			1.000			1.000			1.000
< 60y	18 (23)	60 (77)		36 (46)	42 (54)		32 (41)	46 (59)		12 (15)	66 (85)		33 (42)	45 (58)	
≥ 60y	5 (23)	17 (77)		8 (36)	14 (64)		10 (45)	12 (55)		2 (9)	20 (91)		9 (41)	13 (59)	
Tumor size			1.000			1.000			1.000			1.000			1.000
< 15 cm	19 (26)	55 (74)		33 (45)	41 (55)		32 (43)	42 (57)		10 (13)	64 (87)		31 (42)	43 (58)	
≥ 15 cm	4 (15)	22 (85)		11 (42)	15 (58)		10 (38)	16 (62)		4 (15)	22 (85)		11 (42)	15 (58)	
Laterality			1.000			1.000			1.000			1.000			1.000
Unilaterality	17 (22)	62 (78)		35 (44)	44 (56)		32 (40)	47 (60)		12 (15)	67 (85)		34 (43)	45 (57)	
Bilaterality	6 (29)	15 (71)		9 (43)	12 (57)		10 (48)	11 (52)		2 (9)	19 (91)		8 (38)	13 (62)	
CA125			1.000			1.000			1.000			1.000			1.000
Normal	4 (22)	14 (78)		6 (33)	12 (67)		6 (33)	12 (67)		3 (17)	15 (83)		8 (44)	10 (56)	
Abnormal	16 (25)	48 (75)		28 (44)	36 (56)		28 (44)	36 (56)		10 (16)	54 (84)		24 (38)	40 (62)	
Ascites			1.000			1.000			1.000			1.000			1.000
Absent	11 (21)	42 (79)		25 (47)	28 (53)		20 (38)	33 (62)		9 (17)	44 (83)		26 (49)	27 (51)	
Present	12 (25)	35 (75)		19 (40)	28 (60)		22 (47)	25 (53)		5 (11)	42 (89)		16 (34)	31 (66)	
Endometriosis			1.000			1.000			1.000			1.000			1.000
Absent	16 (25)	48 (75)		30 (47)	34 (53)		30 (47)	34 (53)		8 (12)	56 (88)		27 (42)	37 (58)	
Present	7 (19)	29 (81)		14 (39)	22 (61)		12 (33)	24 (67)		6 (17)	30 (83)		15 (42)	21 (58)	
Stage			1.000			1.000			1.000			1.000			1.000
I-II	13 (19)	56 (81)		28 (41)	41 (59)		27 (39)	42 (61)		8 (12)	61 (88)		28 (41)	41 (59)	
III-IV	10 (32)	21 (68)		16 (52)	15 (48)		15 (48)	16 (52)		6 (19)	25 (81)		14 (45)	17 (55)	
Disease Progression			1.000			1.000			1.000			1.000			1.000
No	12 (22)	42 (78)		24 (44)	30 (56)		22 (41)	32 (59)		6 (11)	48 (89)		22 (41)	32 (59)	
Yes	11 (24)	35 (76)		20 (44)	26 (56)		20 (43)	26 (57)		8 (17)	38 (83)		20 (44)	26 (56)	
Survival Status			1.000			1.000			1.000			1.000			1.000
Alive	13 (21)	49 (79)		28 (45)	34 (55)		27 (43)	35 (57)		7 (11)	55 (89)		27 (44)	35 (56)	
Dead	10 (26)	28 (74)		16 (42)	22 (58)		15 (39)	23 (61)		7 (18)	31 (82)		15 (40)	23 (60)	
pAKT			**0.020**			** < 0.001**			0.552			1.000			
Negative	17 (41)	25 (59)		32 (76)	10 (24)		23 (55)	19 (45)		8 (19)	34 (81)				
Positive	6 (10)	52 (90)		12 (21)	46 (79)		19 (33)	39 (67)		6 (10)	52 (90)				
PIK3CA Exon9			1.000			1.000			**0.035**			1.000			1.000
Wild-type	14 (30)	32 (70)		19 (41)	27 (59)		29 (63)	17 (37)		7 (15)	39 (85)		17 (37)	29 (63)	
Mutant	5 (17)	24 (83)		18 (62)	11 (38)		7 (24)	22 (76)		6 (21)	23 (79)		15 (52)	14 (48)	
PIK3CA Exon20			1.000			1.000			1.000			1.000			1.000
Wild-type	16 (26)	45 (74)		31 (51)	30 (49)		29 (47)	32 (53)		11 (18)	50 (82)		28 (46)	33 (54)	
Mutant	3 (19)	13 (81)		7 (44)	9 (56)		8 (50)	8 (50)		2 (12)	14 (88)		5 (31)	11 (69)	

Survival analyses demonstrated that patients with early-stage disease (FIGO I–II), unilateral tumors and absence of ascites had significantly better OS and PFS than those with advanced-stage disease, bilateral involvement and ascites (Supplementary Fig. 1 and 2a,b,d). Elevated preoperative CA125 levels were associated with poorer PFS, although no significant association with OS was observed (Supplementary Fig. 1c and 2c). Notably, analysis of individual PI3K catalytic subunits in the overall cohort did not reveal significant associations with OS or PFS (Supplementary Fig. 1 and 2e–o), suggesting that the prognostic impact of PI3K signaling may be context-dependent.

### Co-expression architecture of PI3K catalytic isoforms reveals heterogeneous signaling patterns

Considering the context-dependent redundancy and compensatory interactions among PI3K catalytic isoforms [8–10], we next investigated the combinatorial expression patterns of p110α, p110β, p110δ and p110γ rather than evaluating each isoform independently.

Among the 100 evaluable tumors, the most common expression pattern was simultaneous positivity for all four catalytic subunits, observed in 35% of cases. Isolated expression of individual isoforms was relatively uncommon and was identified in only 1% of tumors for p110α and 12% for p110γ. Additional co-expression patterns included p110α + p110β + p110γ (12%), p110α + p110δ + p110γ (11%), p110α + p110γ (9%), p110α + p110δ (5%), p110β + p110γ (3%), p110α + p110β + p110δ (3%), p110δ + p110γ (2%), p110β + p110δ + p110γ (2%), and p110α + p110β (1%). Approximately 4% of tumors showed no detectable expression of PI3K catalytic subunits.

These findings revealed marked inter-patient heterogeneity in PI3K isoform co-expression patterns. Importantly, several low-frequency isoforms rarely occurred alone but preferentially co-expressed with dominant isoforms, suggesting potential cooperative or compensatory signaling interactions within the PI3K pathway.

We further analyzed the relationship between PI3K co-expression patterns and pAKT activation (Table 2). Notably, simultaneous expression of all four catalytic subunits was significantly associated with pAKT positivity (p < 0.001). No statistically significant associations were identified for other expression combinations.

**Table 2:** Association of PI3K catalytic subunit expression with pAKT expression

Parameters	pAKT		
	Negative(%)	Positive(%)	P
p110α			0.607
Negative	41(41)	58(59)	
Positive	1(100)	0(0)	
p110γ			0.201
Negative	33(38)	55(62)	
Positive	9(75)	3(25)	
p110α + p110β			1
Negative	42(42)	57(58)	
Positive	0(0)	1(100)	
p110α + p110δ			0.841
Negative	39(41)	56(59)	
Positive	3(60)	2(40)	
p110α + p110γ			0.377
Negative	36(40)	55(60)	
Positive	6(67)	3(33)	
p110β + p110γ			1
Negative	41(42)	56(58)	
Positive	1(33)	2(67)	
p110δ + p110γ			0.377
Negative	40(41)	58(59)	
Positive	2(100)	0(0)	
p110α + p110β + p110δ			1
Negative	41(42)	56(58)	
Positive	1(33)	2(67)	
p110α + p110β + p110γ			0.548
Negative	39(44)	49(56)	
Positive	3(25)	9(75)	
p110α + p110δ + p110γ			0.211
Negative	34(38)	55(62)	
Positive	8(73)	3(27)	
p110β + p110δ + p110γ			0.377
Negative	40(41)	58(59)	
Positive	2(100)	0(0)	
p110α + p110β + p110δ + p110γ			** < 0.001**
Negative	39(60)	26(40)	
Positive	3(9)	32(91)	
none			0.548
Negative	39(41)	57(59)	
Positive	3(75)	1(25)	

^*^Categories with very small sample sizes (n ≤ 3) were considered exploratory, and the corresponding statistical comparisons should be interpreted with caution

### Prognostic significance of PI3K pathway activation in advanced-stage OCCC

Given that advanced-stage OCCC is characterized by aggressive biological behavior and that PI3K pathway dysregulation is common in OCCC [11, 12], we further investigated the prognostic significance of PI3K signaling specifically in patients with FIGO stage III–IV disease.

**Fig. 3:**
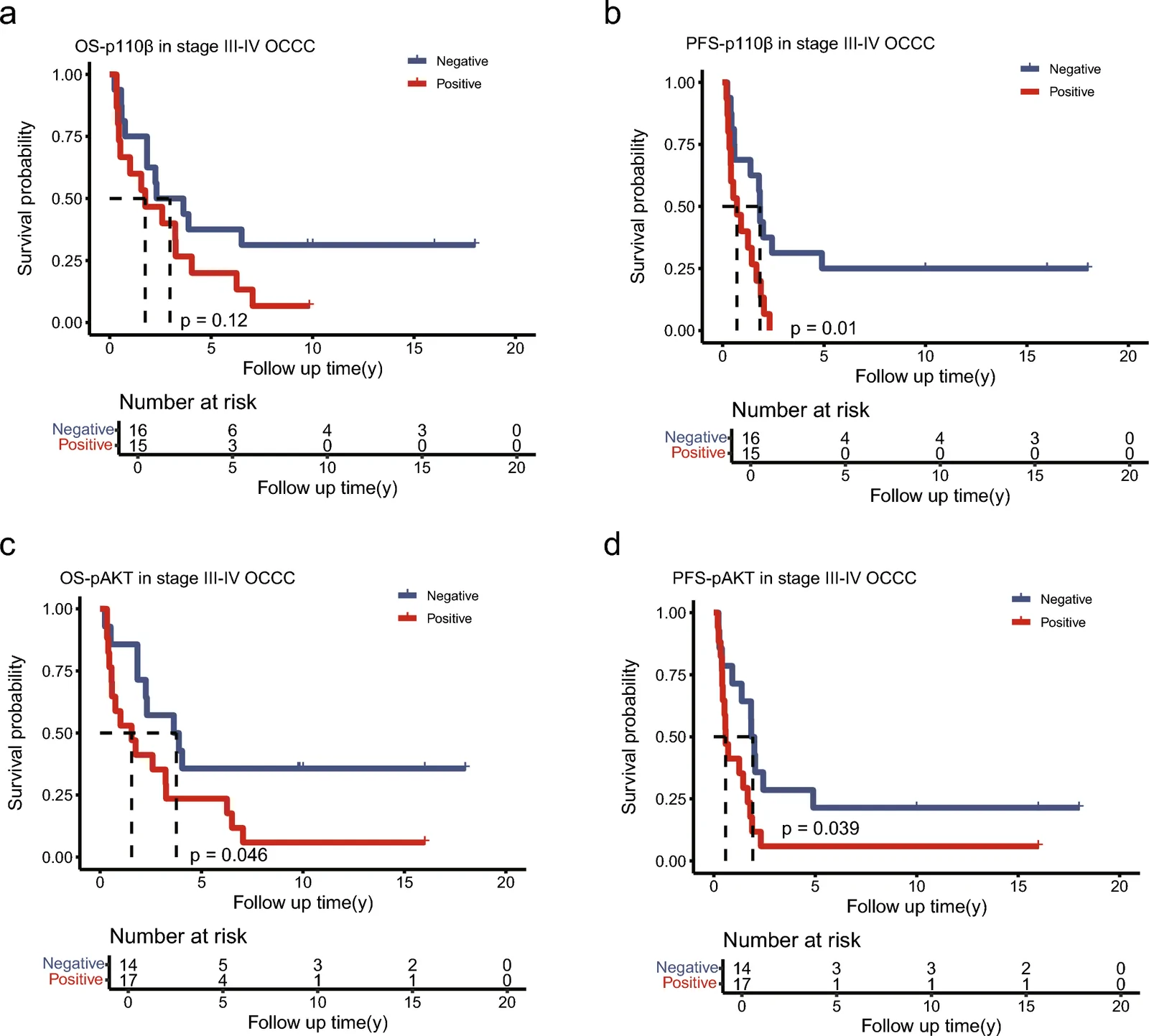
Kaplan–Meier survival curves of (**a**) the expression of p110β for OS in stage III-IV OCCC, **b** the expression of p110β for PFS in stage III-IV OCCC, **c** the expression of pAKT for OS in stage III-IV OCCC, **d** the expression of pAKT for PFS in stage III-IV OCCC

In advanced-stage tumors, p110β expression was significantly associated with poorer PFS (p = 0.01). Moreover, patients with p110β-positive tumors exhibited shorter OS numerically than the p110β-negative group, although the difference did not reach statistical significance (log-rank p = 0.12) (Fig. 3a, b).

Furthermore, pAKT positivity was significantly associated with both shorter OS (p = 0.046) and poorer PFS (p = 0.039) (Fig. 3c, d). In contrast, other clinicopathological parameters did not significantly affect survival outcomes in advanced-stage cases (Supplementary Fig. 3a–i).

Univariate Cox regression analysis identified advanced-stage (HR = 8.762, p < 0.001), bilateral involvement (HR = 4.213, p < 0.001), and the presence of ascites (HR = 2.403, p = 0.009) as factors associated with poorer OS. Similarly, univariate analysis for PFS demonstrated that advanced-stage (HR = 6.887, p < 0.001), bilateral involvement (HR = 3.624, p < 0.001), ascites (HR = 1.969, p = 0.024), and elevated CA125 levels (HR = 3.065, p = 0.035) were significantly associated with unfavorable prognosis. Multivariate Cox regression analysis further confirmed advanced stage as an independent prognostic factor for both worse OS (HR = 6.687, p < 0.001) and poorer PFS (HR = 5.356, p < 0.001) (Table 3).

**Table 3:** Univariable and multivariable cox regression analysis of clinicopathological characteristics

Parameters	PFS			OS		
	HR	CI(95%)	P	HR	CI(95%)	P
Univariable						
Age						
Tumor size						
Laterality	3.624	1.980–6.634	** < 0.001**	4.213	2.179–8.146	** < 0.001**
CA125	3.065	1.082–8.678	**0.035**			
Ascites	1.969	1.095–3.543	**0.024**	2.403	1.242–4.647	**0.009**
Endometriosis						
Stage	6.887	3.761–12.609	** < 0.001**	8.762	4.422–17.360	** < 0.001**
p110α						
p110β						
p110δ						
p110γ						
pAKT						
PIK3CA Exon9						
PIK3CA Exon20						
Multivariable						
Laterality	1.3	0.579–2.921	0.525	1.667	0.801–3.466	0.172
CA125	2.165	0.722–6.488	0.168			
Ascites	1.019	0.456–2.278	0.963	1.263	0.621–2.566	0.519
Stage	5.356	2.443–11.743	** < 0.001**	6.687	3.070–14.567	** < 0.001**

To further evaluate the prognostic significance of PI3K signaling in patients with advanced-stage disease (FIGO stage III–IV), we performed additional univariate Cox regression analysis. The results showed that p110β expression (HR = 2.800, 95% CI: 1.235–6.348, p = 0.014) and pAKT expression (HR = 2.268, 95% CI: 1.022–5.030, p = 0.044) were significantly associated with poorer PFS in this subgroup (Table 4).

**Table 4:** Univariate cox regression analysis of prognostic factors in patients with advanced-stage disease

Parameters	PFS			OS		
	HR	CI(95%)	P	HR	CI(95%)	P
Univariable						
Age						
Tumor size						
Laterality						
CA125						
Ascites						
Endometriosis						
Stage						
p110α						
p110β	2.8	1.235–6.348	**0.014**			
p110δ						
p110γ						
pAKT	2.268	1.022–5.030	**0.044**			
PIK3CA Exon9						
PIK3CA Exon20						

Collectively, these findings indicate that although individual PI3K catalytic subunits have limited prognostic value in the overall OCCC cohort, activation of the PI3K pathway—particularly pAKT signaling—identifies a biologically aggressive subset of advanced-stage tumors associated with poor clinical outcomes. In addition, p110β expression was associated with shorter PFS in patients with advanced-stage disease, suggesting that p110β-mediated PI3K signaling may contribute to disease progression in this subgroup.

## Discussion

The PI3K/AKT/mTOR (PAM) signaling pathway plays a central role in regulating cell proliferation, survival, and apoptosis, and its dysregulation is a well-established driver of tumorigenesis. As a lipid kinase, PI3K initiates intracellular signaling cascades that regulate diverse cellular processes, including proliferation, metabolism, migration, and survival [13, 14]. Among PI3K family members, class I PI3Ks are activated by growth factor signaling and function as heterodimers consisting of a regulatory subunit (p85) and a catalytic subunit (p110) [15]. Class I PI3Ks comprise four catalytic isoforms: p110α (encoded by PIK3CA), p110β (encoded by PIK3CB), p110δ (encoded by PIK3CD), and p110γ (encoded by PIK3CG) [16, 17].

Emerging evidence suggests that different PI3K isoforms exert distinct and context-dependent roles in oncogenic signaling and tumor progression [17–19]. Among them, p110α is the best-characterized isoform and is frequently altered through PIK3CA mutations across multiple cancer types, serving as a major oncogenic driver in PIK3CA-dependent tumors [20]. By contrast, p110β, p110δ, and p110γ are less frequently mutated but may still contribute to aberrant PI3K signaling through alternative mechanisms, including immune signaling and inflammatory responses [21–23]. OCCC is characterized by frequent alterations in the PAM pathway, with previous studies primarily focusing on PIK3CA mutations and p110α dysregulation [24–26]. However, the expression patterns and clinical significance of other class I PI3K catalytic subunits in OCCC remain largely unexplored. In the present study, we found that p110α and p110γ were the predominant isoforms expressed in OCCC. Notably, the class I PI3K catalytic subunits p110α and p110β were positively associated with pAKT activation, whereas p110δ and p110γ showed no significant association. These findings are consistent with prior studies suggesting that elevated class I PI3K activity is associated with enhanced AKT phosphorylation in multiple malignancies [27–29]. Beyond individual isoforms, we observed frequent co-expression of multiple PI3K catalytic subunits, with the four-subunit pattern (p110α + p110β + p110δ + p110γ) representing the predominant expression profile. Such co-expression patterns may reflect pathway redundancy and sustained PI3K signaling activity in OCCC [8, 30, 31]. The simultaneous expression of multiple isoforms may enable functional cooperation within the PI3K network, allowing tumor cells to maintain signaling robustness under heterogeneous genetic and microenvironmental conditions [32]. This signaling redundancy may partly explain the limited efficacy of single-isoform inhibition and supports the potential rationale for broader or combination PI3K-targeted therapeutic strategies [9, 30, 33].

Among non-α isoforms, p110β has been increasingly recognized as a critical mediator of cell survival, migration, and therapeutic resistance, particularly in PTEN-deficient and EGFR-driven tumors [34, 35]. Importantly, we identified p110β as a potential prognostic factor in advanced-stage OCCC, where elevated p110β expression was associated with poorer PFS. Similarly, increased pAKT activation was associated with both poorer OS and PFS in the advanced-stage subgroup, further supporting the biological relevance of PI3K pathway activation in aggressive disease. As a downstream effector of the PI3K pathway, elevated pAKT expression has been associated with poor prognosis in several hematologic and solid tumors [36]. Overexpression of p110β mRNA in colorectal cancer is associated with shorter disease-free survival [29], while high expression levels of all four isoforms (p110α, p110β, p110δ, p110γ) correlate with poor prognosis in diffuse large B-cell lymphoma [37]. Collectively, these findings suggest that p110β expression may serve as a prognostic biomarker for disease progression in advanced-stage OCCC, whereas pAKT activation may represent a broader indicator of poor survival outcomes. Furthermore, these observations support the potential therapeutic relevance of PI3K pathway inhibition in OCCC. Nevertheless, further mechanistic studies and multicenter validation studies are warranted to determine whether isoform-specific or combination PI3K inhibition could improve clinical outcomes in patients with OCCC.

This study has several limitations that should be acknowledged. First, this was a retrospective single-center study with a relatively limited sample size, and some clinicopathological variables were not systematically recorded in historical cases due to the retrospective nature of the study, which may limit the completeness of pathological characterization. Given the rarity of OCCC, validation in larger, multicenter cohorts will be necessary to confirm the prognostic significance of PI3K isoform expression. Second, due to prolonged storage of FFPE tissue blocks, PIK3CA mutational analysis could not be completed in all cases, which may have reduced the statistical power for assessing associations between genomic alterations and PI3K isoform expression. In addition, although immunohistochemistry enabled systematic evaluation of PI3K catalytic subunit expression and pAKT activation, protein expression does not necessarily reflect functional signaling activity. Therefore, mechanistic studies are required to determine whether the observed co-expression patterns directly contribute to pathway redundancy, tumor progression, or therapeutic resistance. Third, several PI3K co-expression patterns occurred at relatively low frequencies, limiting statistical robustness and increasing the possibility of overestimating prognostic associations in subgroup analyses. Moreover, the number of patients with stage III–IV disease was relatively small, and only a few patients remained at risk during long-term follow-up, which may have resulted in unstable Kaplan–Meier survival estimates at later time points. Therefore, survival estimates for the tails of the curves and subgroup-specific prognostic associations should be interpreted with caution and further validated in larger cohorts. Finally, while our findings suggest potential therapeutic implications for broader or combination PI3K inhibition, the present study was observational and did not directly evaluate treatment responses. Accordingly, the proposed therapeutic relevance of specific PI3K isoforms remains hypothesis-generating and warrants further experimental and clinical validation.

In summary, our study provides a comprehensive characterization of class I PI3K catalytic subunit expression patterns in OCCC and highlights substantial heterogeneity in isoform distribution and co-expression. We found that the class I PI3K catalytic subunits, p110α and p110β, were positively associated with pAKT activation. Importantly, high p110β expression was associated with poorer PFS in patients with advanced-stage OCCC, whereas increased pAKT activation was associated with both poorer OS and PFS in this subgroup. Moreover, both high p110β expression and increased pAKT activation were identified as adverse prognostic factors for PFS in advanced-stage disease. Together, these findings support the biological importance of PI3K signaling heterogeneity in OCCC and provide a rationale for further investigation of isoform-specific and combination PI3K-targeted strategies in larger prospective studies.

## Supplementary Information

Below is the link to the electronic supplementary material.

Supplementary file1 (PDF 1020 KB)

Supplementary file2 (PDF 1014 KB)

Supplementary file3 (PDF 744 KB)

Supplementary file4 (XLSX 11 KB)

Supplementary file5 (XLSX 10 KB)

Supplementary file6 (XLSX 9 KB)

Supplementary file7 (XLSX 11 KB)

## Data Availability

The datasets generated during and/or analysed during the current study are available from the corresponding author on reasonable request.
