# Supplementary material for: PIK3CA mutations and PI3K isoform expression define prognostic signatures in ovarian clear cell carcinoma
Source: Arch Gynecol Obstet. 2026 Jul 14;313(1):286. doi: 10.1007/s00404-026-08525-w (PMC13645935; doi:10.1007/s00404-026-08525-w)
Supplement: Supplementary file 1 [file 404_2026_8525_MOESM1_ESM.pdf]

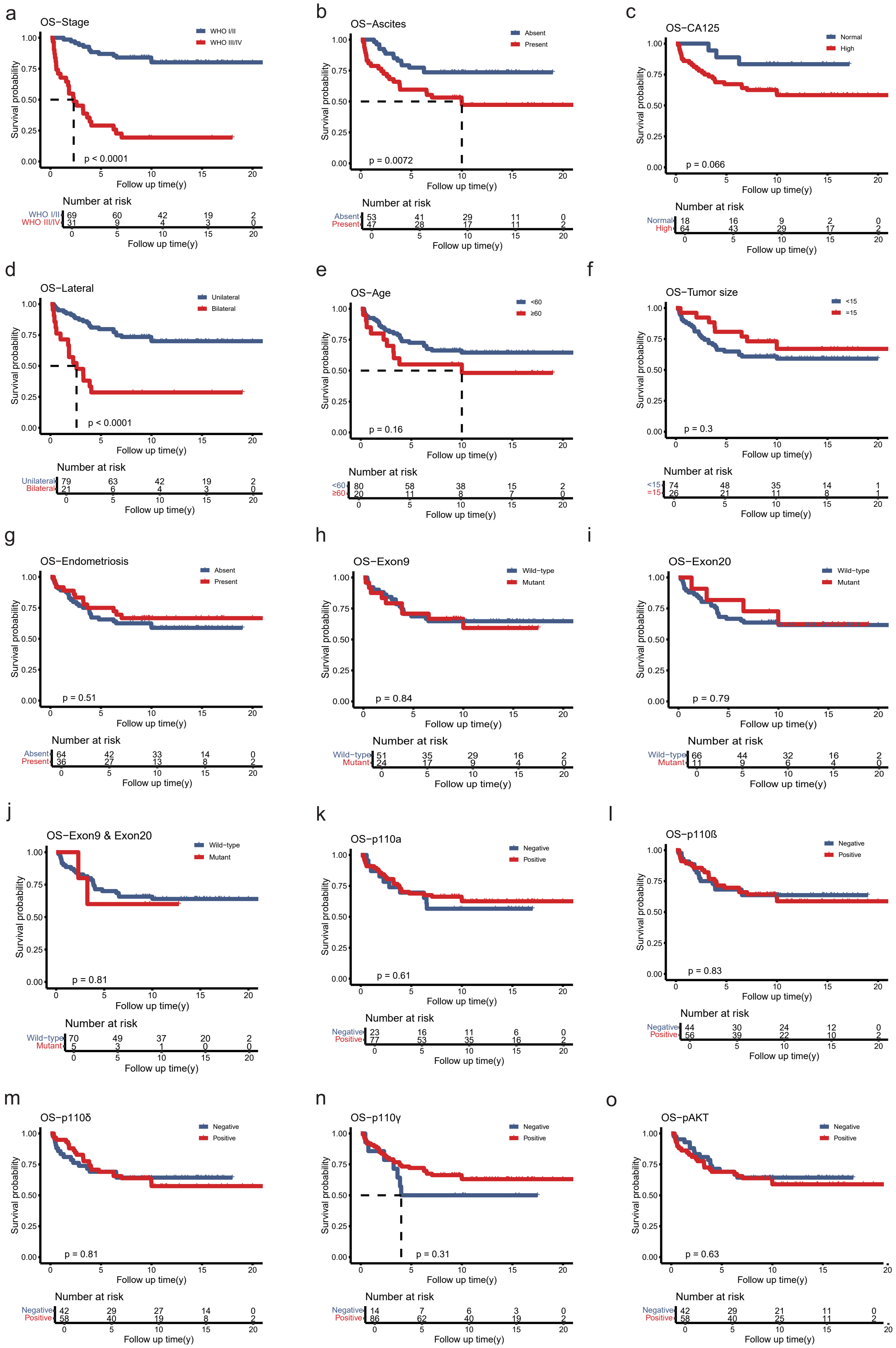

Supplementary Figure 1 Kaplan-Meier survival curves for OS of ovarian clear cell carcinoma(OCCC) are shown for (a) FIGO Stage, (b) the history of ascites, (c) the preoperative serum CA125 level, (d) tumor laterality, (e) age, (f) tumor size, (g) the history of endometriosis, (h) the mutation in PIK3CA exon 9, (i) the mutation in PIK3CA exon 20, (j) dual mutations in PIK3CA exon 9 and 20, (k) the expression of p110α, (l) the expression of p110β, (m) the expression of p110δ, (n) the expression of p110γ, (o) the expression of pAKT.
