# Supplementary material for: PIK3CA mutations and PI3K isoform expression define prognostic signatures in ovarian clear cell carcinoma
Source: Arch Gynecol Obstet. 2026 Jul 14;313(1):286. doi: 10.1007/s00404-026-08525-w (PMC13645935; doi:10.1007/s00404-026-08525-w)
Supplement: Supplementary file 3 [file 404_2026_8525_MOESM3_ESM.pdf]

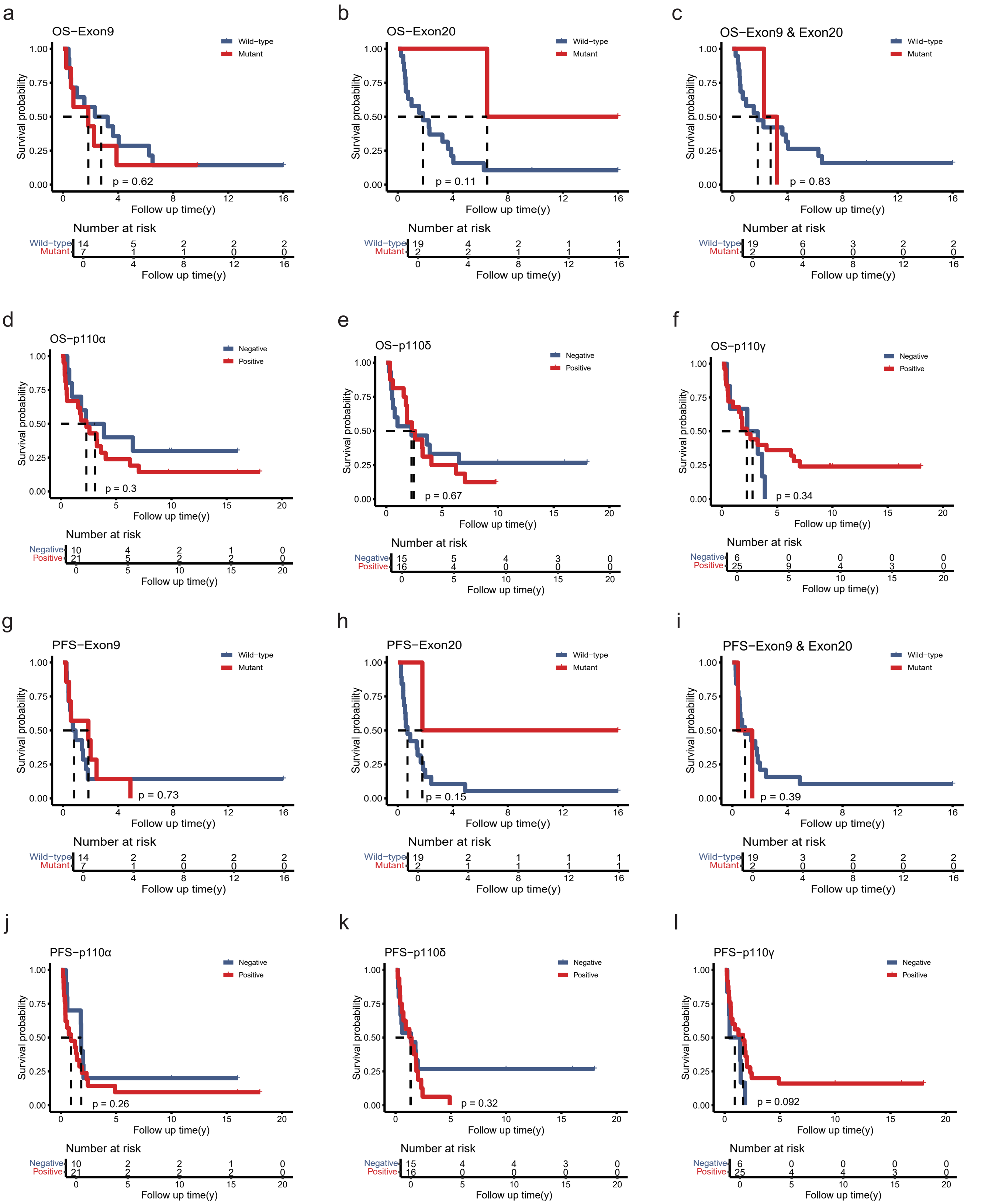

Supplementary Figure 3 Kaplan-Meier survival curves in patients with advanced OCCC are shown for (a) OS of the mutation in PIK3CA exon 9, (b) OS of the mutation in PIK3CA exon 20, (c) OS of the dual mutations in PIK3CA exon 9 and 20, (d) OS of the expression of p110α, (e) OS of the expression of p110 δ, (f) OS of the expression of p110γ, (g) PFS of the mutation in PIK3CA exon 9, (h) PFS of the mutation in PIK3CA exon 20, (i) PFS of the dual mutations in PIK3CA exon 9 and 20, (j) PFS of the expression of p110α, (k) PFS of the expression of p110δ, (l) PFS of the expression of p110γ.
